# A survey of human shoulder functional kinematic representations

**DOI:** 10.1007/s11517-018-1903-3

**Published:** 2018-10-26

**Authors:** Rakesh Krishnan, Niclas Björsell, Elena M. Gutierrez-Farewik, Christian Smith

**Affiliations:** 1 Department of Electronics, Mathematics and Natural Sciences, University of Gävle, Gävle, Sweden; 2 Robotics, Perception and Learning (RPL), School of Computer Science and Communication, Royal Institute of Technology (KTH), Stockholm, Sweden; 3 KTH Engineering Sciences, Mechanics, Royal Institute of Technology (KTH), Stockholm, Sweden; 4 BioMEx Center, Royal Institute of Technology (KTH), Stockholm, Sweden

**Keywords:** Kinematics, Robot-assisted rehabilitation, Human movement understanding, Human-robot interaction, Shoulder

## Abstract

In this survey, we review the field of human shoulder functional kinematic representations. The central question of this review is to evaluate whether the current approaches in shoulder kinematics can meet the high-reliability computational challenge. This challenge is posed by applications such as robot-assisted rehabilitation. Currently, the role of kinematic representations in such applications has been mostly overlooked. Therefore, we have systematically searched and summarised the existing literature on shoulder kinematics. The shoulder is an important functional joint, and its large range of motion (ROM) poses several mathematical and practical challenges. Frequently, in kinematic analysis, the role of the shoulder articulation is approximated to a ball-and-socket joint. Following the high-reliability computational challenge, our review challenges this inappropriate use of reductionism. Therefore, we propose that this challenge could be met by kinematic representations, that are redundant, that use an active interpretation and that emphasise on functional understanding.

## Introduction

Human movement is in the spotlight as researchers attempt to design and successfully interface machines with humans. Importantly, the success of these devices relies on the interaction design. Equivalently, the reliable parameterisation of human movement is important in generating computer models in biomechanics. Although human movement kinematics is of central importance in both these fields, the underlying level of abstraction, detail and purpose are diverse. Here, the fundamental difference lies in the underlying mechanisms. Robot motion can often be modelled repeatably using simplified laws of physics, such as pure rotational joints. In contrast, such laws cannot completely and reliably describe biological motion [1]. Therefore, this review aims not only to classify and summarise the existing literature but also to draw attention towards several knowledge gaps in movement kinematics in general and shoulder kinematics in particular.

### Need for a review

Reviewing shoulder kinematics is challenging due to the functional complexity [2], diversity of objectives, diversity in kinematic representations and protocols used in the literature [3–5]. Traditionally, in biomechanics, 3D motion analysis has been used in the qualitative and quantitative evaluation of biological health [6]. In human motor control, kinematics is used to understand the underlying neural policy [3]. Although human movement has been studied in biomechanics and motor control for several decades, it is only recently that human movement has emerged as a mainstream research topic in robotics [7, 8]. Current trends in robotics research are moving towards the concept of human-centric models. Such models are based on a functional understanding of humans and have the potential to act as templates for developing technology that can improve the end goals of a rehabilitation intervention [9].

Despite this need, there is a lack of up-to-date literature on functional shoulder kinematics. To the best of our knowledge, the only available review on this topic was published by Maurel and Thalmann [10], in which the main focus was on dynamic simulation. Note that in such applications, the interest is in describing and reproducing observed movements. Such an analysis is not of immediate help in human-robot interaction (HRI).

### Role of movement kinematics in HRI

In HRI, a key bottleneck exists as to how the robot can understand the movement cues from the human user [11]. Without this essential knowledge, the robot cannot operate in synchrony with the human, thus raising concerns of usability and safety [11]. Estimating human intention from the brain signals or muscles is computationally daunting. However, kinematics has the potential to be the primary level of understanding intention because the higher we climb the ladder of motor hierarchies, the greater the level of abstraction of the intention signals is [1]. However, even if kinematics can be used as an implicit command, there is no agreement on the mathematical framework that is most suitable for this purpose [12–18].

Currently, the majority of HRI review papers cover only the physical aspects [18–20]. In fact, it is the cognitive interaction that in turn drives the physical HRI [21]. Mainly, in cognitive HRI (cHRI), such as in robot-assisted rehabilitation, there is an active knowledge-based two-way dialogue between the human user and the robot [22]. In such an advanced HRI problem, kinematics is essential in the steps of intention modelling, design, reasoning, planning, execution and user evaluation [9, 21, 23–25].

In HRI, replicating 3D upper arm kinematics is a challenge [12, 13]. Understanding the principles of the human upper limb poses a non-trivial computational problem; overall, there is a lack of reliable tools and evaluation metrics for this purpose [3, 12, 14]. In recent years, there have been strong criticisms against the validity of “the promise of robot-assisted rehabilitation” (see [26]). Thus far, robot-assisted rehabilitation has been able to demonstrate its real benefits only at a kinematic level [27]. Despite these promising results, many of the existing robotic solutions oversimplify the upper limb kinematics [23].

### Aims and scope

In this review, we aim to summarise the existing literature on functional shoulder kinematics. Because this topic is interdisciplinary, we attempt to integrate the knowledge from several diverse research communities. Importantly, in rehabilitation technology, it is expected that the robotic solutions yield consistent results [28]. Therefore, it is a pre-requisite that the computational framework which drives the HRI be highly reliable [28]. In the future, we hope that the findings of our review will be translated into effective robot-assisted rehabilitative solutions like exoskeletons. Primarily, this technology aims for functional compensation or assistance [29, 30]. Therefore, we limit our review to papers addressing functional shoulder kinematics.

To clarify, a “functional shoulder” is gauged by painlessness, mobility, a harmonious motion pattern between the joints, and stability [31, 32]. In this review, function implies that the emphasis is on the day-to-day use of the shoulder. Although the focus is on functional kinematic representations, we briefly mention other existing literatures wherever relevant.

### Role of kinematic representations

Kinematic representations can be thought of as mathematical structures that model the movement of interest. Different kinematic representations are helpful in extending and updating our understanding of various underlying mechanisms of the neuromuscular system [33]. Note that their choice is not unique; rather, it is context- or application-specific [34, 35].

What is the high-reliability requirement in shoulder kinematics? The answer can be divided possibly into three parts. First, when using kinematic representations, numerical singularities pose the problem of ambiguity, which in turn might lead to ambiguity in the volitional command that drives HRI. Such a situation must be avoided at any cost. Therefore, a lack of numerical singularities is paramount.

Second, when movement variability is used to understand the underlying neural policy, computational reliability is very important. A compromise in this regard can undermine the conclusions of the study [36, 37]. Mainly, for the same movement, a different choice of kinematic representations can result in conflicting results [38]. This fact is often overlooked in robotics. In robotics, interest has been limited to finding a consistent and repeatable solution with no element of causation or reasoning in mind [8, 39].

Third, the mathematical representation must faithfully follow the physiological kinematics [37]. A violation of this requirement results in a representational mismatch. This error is usually small for joints with small range of motion (ROM). However, because the shoulder is one of the joints with the largest ROM, this error would be very high. Therefore, we critically evaluate the existing literature in light of this high-reliability computational challenge.

Our review opens with a description of human shoulder anatomy and basic shoulder movements (see Section 2). This description is followed by a section on the challenges involved in shoulder kinematics (see Section 3 ). This is followed by the review search strategy, outline, classification and summary (see Section 5). This section is supplemented by a discussion in Section 6. Finally, we present possible research directions that can meet the high-reliability computational challenge (see Section 7).

## Functional anatomy and movements

A functional shoulder is a pre-requisite for good upper arm functioning, as it places, operates and controls the forearm [40]. Without the active and significant contribution of the human shoulder, many daily living activities like hair combing and reaching the back cannot be performed successfully. Importantly, the musculoskeletal system provides the basis for constraining and allowing movement. This ability to generate movement is dependent on the structural morphology, which is studied under the realm of functional anatomy. Understanding the functional anatomy provides insight into the working aspects of any complex joint. Note that the muscular system and the structure of the various joint capsules are outside the scope of this paper.

### Bones and joints

**Fig. 1:**
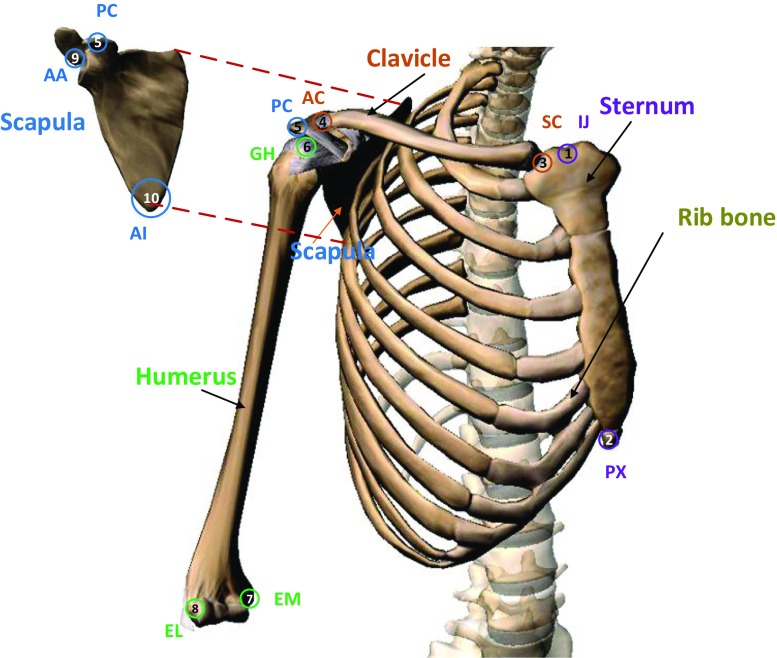
Anterior view of right shoulder with the International Society of Biomechanics (ISB)-recommended bony landmarks: 1 incisura jugularis (IJ), 2 processus xiphoideus (PX), 3 sternoclavicular joint (SC), 4 acromioclavicular joint (AC), 5 processus coracoideus (PC), 6 glenohumeral joint (GH), 7 medial epicondyle (EM), 8 lateral epicondyle (EL), 9 angulus acromialis (AA), 10 angulus inferior (AI) (image courtesy: Visible Body Skeleton premium)

**Fig. 2:**
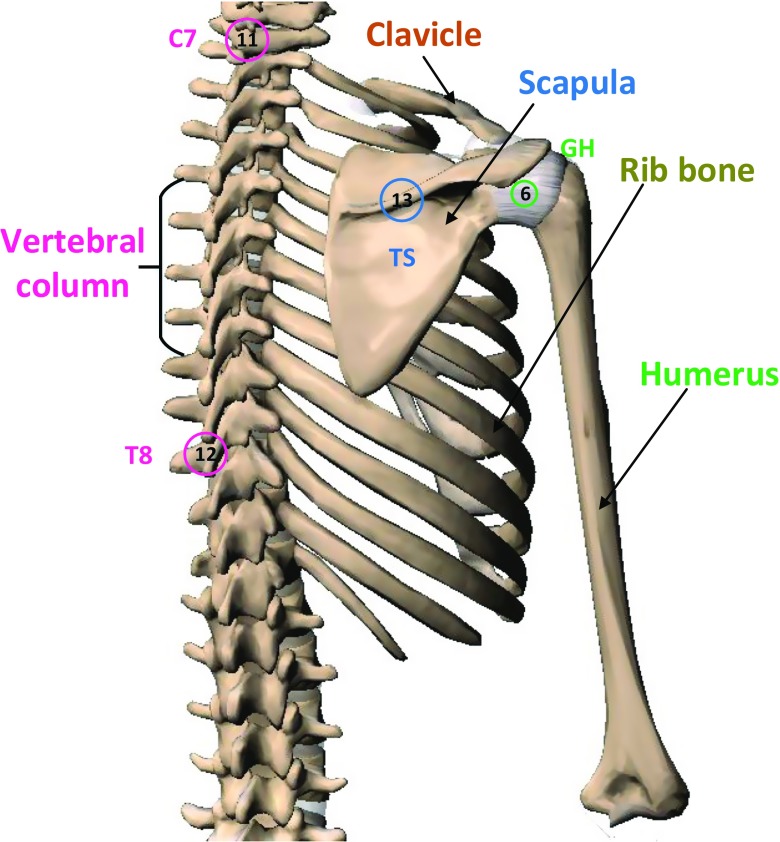
Posterior view of the right shoulder with International Society of Biomechanics (ISB)-recommended bony landmarks: 6 glenohumeral joint (GH), 11 processus spinous 7th cervical vertebra (C7), 12 processus spinous 8th thoracic vertebra (T8), 13 trigonum spinae scapulae (TS) (image courtesy: Visible Body Skeleton premium)

Bones are primarily rigid structures that form the supportive base for the muscles to act on. The kinematic role of the bone is approximated by straight-line distances between end-points known as links [31]. A detailed illustration of the shoulder articulation from the anterior and posterior views with labelled bony landmarks is shown in Figs. 1 and 2. The shoulder kinematic chain starts from the *sternum*, the chest bone that constitutes the midline of the anterior thorax. The sternum is followed by the S-shaped collar bone, known as the *clavicle*. The mechanical action of the clavicle is like that of a crankshaft [5, 31, 41].

The third bone that forms the shoulder girdle is the flat posteriorly located bone known as the *scapula*. The positioning of the scapula in turn depends on the hand usage and loading [40]. The glenoid cavity of the scapula acts as the site of attachment for the upper arm bone called the *humerus*. This attachment to the glenoid is mainly achieved through the spherical head of the humerus.

The joints are the meeting surfaces of the bones. There are three synovial joints in the shoulder. The interface between the sternum and the proximal end of the clavicle forms the *sternoclavicular* (SC) joint. The distal end of the clavicle connects with the acromion process of the scapula, forming the *acromioclavicular* (AC) joint. Furthermore, the humeral head articulates with the glenoid cavity of the scapula, forming the *glenohumeral* (GH) joint. Additionally, the concave anterior surface of the scapula slides over the convex surface of the thoracic cavity by sandwiching a group of soft tissues, forming the *scapulothoracic* (ST) joint. The ST is a functional joint that accounts for one-third of the shoulder ROM [42]. This fictitious joint is often modelled as a fixed [43] or dynamic contact [10, 44, 45]. Functionally, the shoulder girdle can be approximated by a non-existing humerothoracic (HT) joint, which is commonly found in activities of daily living (ADL) studies.

### Basic shoulder movements

Although the joints of the shoulder articulation are capable of individual motions, their actions are not entirely sequential. Instead, they are simultaneous and well coordinated, resulting in the phenomenon of shoulder rhythm [42]. Importantly, the GH joint has the largest ROM among the shoulder joints due to its low bony congruency and capsular laxity [46]. This peculiarity of the shoulder articulation results in a diverse array of movements. Unfortunately, this diversity has resulted in confusion regarding the most suitable nomenclature for these movements. Therefore, we follow [47] as closely as possible.

**Fig. 3:**
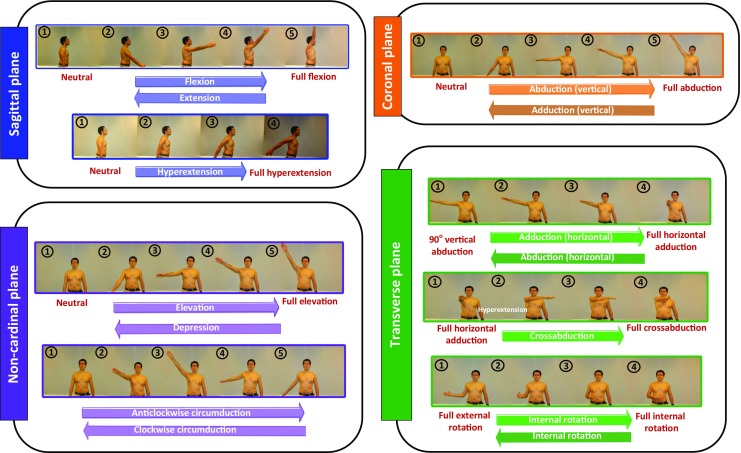
Illustration of various basic shoulder movements

An illustration of different basic shoulder movements is presented in Fig. 3. The shoulder movements in the sagittal plane are called flexion and extension. During *flexion*, the relative humeral angle between the rest position and the fully flexed position varies in the range 0^∘^–180^∘^. The reversal of this motion results in the *extension* phase. If this reversal proceeds posteriorly beyond the neutral position of the humerus, it results in *hyperextension*.

In the coronal plane, movement away from the mid-line of the body is called *abduction*. Similarly, the reverse motion from a fully abducted position to the mid-line is known as *adduction*. The movements in the transverse plane are *internal rotation* and *external rotation*, which constitute the internal or external axial rotation of the humerus. Additionally, the movement of the humerus about the vertical axis results in *horizontal abduction*, *horizontal adduction* and *cross-abduction*, which are unique to the shoulder articulation.

Furthermore, there are movements that are not confined to any cardinal plane (see Fig. 3), namely, the conical movement of the humerus known as *circumduction* and the generalised raising and lowering of the humerus called *elevation* and *depression*.

## Challenges in investigating human shoulder kinematics

There are several challenges in analysing shoulder movement, and they are related to anatomy, function, mathematical description, measurement difficulties or a combination of factors:

- *Complexity*: Human movement is a hierarchical phenomenon wherein the behaviour of the parts does not completely explain the behaviour of the whole, and vice versa [37]. Consequently, single-joint behaviour cannot completely account for multi-joint behaviour [39]. Such a situation makes it difficult to reliably parametrise the upper limb kinematics [48]. The complex anatomy (see Section 2) forces many researchers to limit their analysis to planar motion tasks. It is well known that such kinematic simplifications cannot effectively capture the variety of movements [48, 49].
- *Inconsistent clinical description*: Joint angles defined across the cardinal planes form the basis of human movement analysis. Importantly, the validity of generalised kinematics of rigid bodies depends on the symmetry-preserving properties of the underlying kinematic transformations. Mainly, these symmetry-preserving relationships are mathematically formalised using the notion of the theory of groups [50]. Mathematically, the clinical description does not form a group, which poses mathematical and interpretation difficulties, resulting in controversies such as the Codman paradox [50]. In the shoulder, the actual motions deviate significantly from the clinical description of the cardinal plane motions [6, 46, 48, 51].
- *Measurement limitations*: The large axial rotation of the humerus results in significant soft tissue artefacts (STAs) [4, 48, 50, 52–59], which presents measurement limitations. Recently, a study based on intra-cortical pins successfully quantified the effects of STA on humeral kinematics [60]. Additionally, a study by Naaim et al. [61] compares various multibody optimisation models in STA compensation for different ST joint models. Although this approach is very efficient in minimising the STA, the performance of these group of techniques does depend on the underlying kinematic model [62].
- *Over-constrained system*: Although the individual shoulder bones can move, their motion is often coupled and constrained. This pattern of coupled movement between the shoulder bones is popularly known as shoulder rhythm [63–65]. The extent of this rhythm depends on several aspects, including the plane and arc of elevation, joint anatomy and loading conditions [5, 40].
- *Movement variability*: Variability is an important issue in the literature on human movement. It is a major bottleneck in standardising upper arm kinematics [3]. Moreover, as upper limb movements are discrete, it is challenging to compare the inter-subject and intra-subject kinematics [48]. Movement variability has different origins of two main types: inter-subject and intra-subject variability [37]. Importantly, inter-subject variability has drawn attention and has led to many standardisation initiatives in human shoulder kinematics. The work of the International Shoulder Group (ISG) has led to the well-known International Society of Biomechanics (ISB) coordinate system [66] and an advanced framework [67]. In contrast, such initiatives only partially address the intra-subject variability. Intra-subject variability in movement kinematics is known to emerge from four main factors: representational mismatch, non-standardised protocols, different data processing methods and the actual variability in movement.

## An overview of human shoulder kinematic representations

This section presents a brief review of prominent kinematic representations used to parametrise shoulder movement. We begin with an overview of the relative kinematics problem and present the various mathematical representations used in the literature to address this problem.

### Generalised relative kinematics problem

**Fig. 4:**
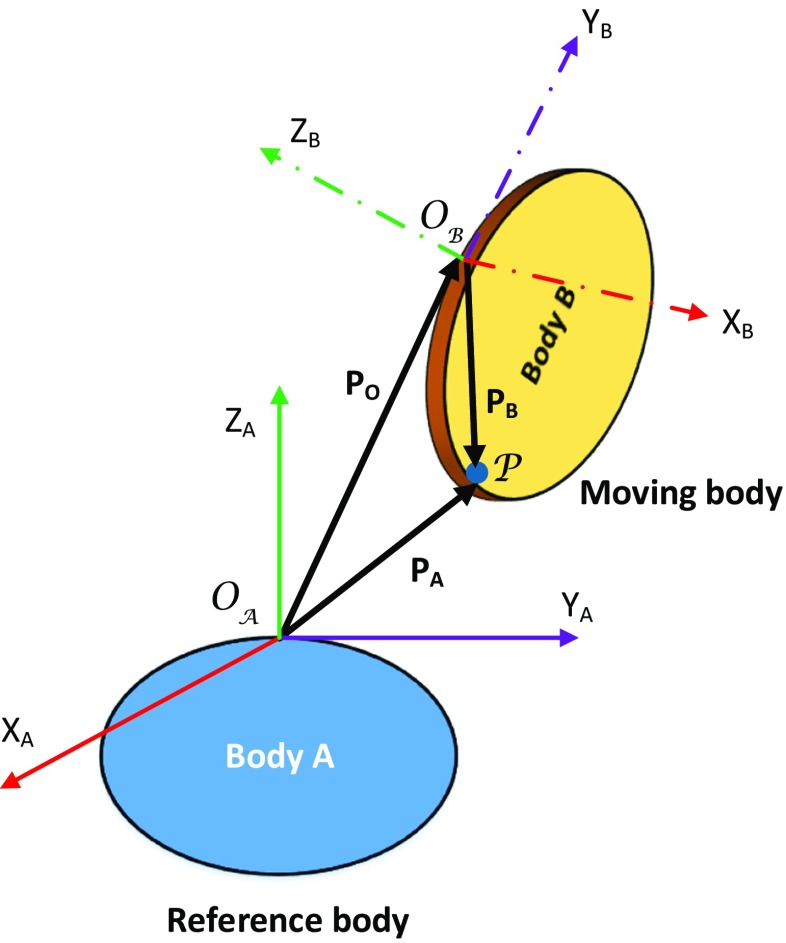
Generalised relative kinematics

As is evident from Section 2.2, the distal segment is always described relative to the proximal segment, which is known as the relative kinematics problem. Consider Fig. 4, a compact way to represent the relative kinematics between the moving body *B* and reference body *A* is given by the homogeneous transformation matrix **T**,
1Here, $\mathbf {R}$ and $\mathbf {t}$ represent the rotation and translation of frame B with respect to frame A, respectively. In human movement, these frames can be defined using anatomical landmarks, mechanical points or axes, or their combination [68]. Note that the interpretation of kinematic data is sensitive to the choice of these frames of reference. In HRI, the robot is equipped with different motion sensors that act both as a measurement system and as a feedback loop. The kinematic representations presented in this section differ in how elements of $\mathbf {T}$ are computed [69]. We present below the prominent kinematic representations in shoulder kinematics below.

### Euler/Cardan angles

Due to the simplicity and intuitive nature of Euler angles, they are very popular in the shoulder kinematics literature. In Euler angles, the rotation matrix $\mathbf {R}$, defined in Eq. 1, is interpreted as a product of three sequential rotational transformations $\mathbf {R_{i}},\mathbf {R_{j}}, \text {and}~\mathbf {R_{k}}$ about the axes $i,j,\text {and}~k$.

2 $$ {\mathbf{R_{(i,j,k)}}}={\mathbf{R_{i}(\theta_{1})R_{j}(\theta_{2})R_{k}(\theta_{3})}} $$

Here, $ {i,j,k}\in \{{X,Y,Z}\} $, provided $ i\neq j,j \neq k$, resulting in 12 different sequences of Euler/Cardan angles. When $ i\neq k$, the resulting asymmetric Euler angles are called Cardan angles [68]. The ISB recommends a symmetric Euler sequence, YXY, for reporting HT kinematics [66].

Although Euler angles are popular due to their intuitive nature, they present limitations due to their numerical instabilities, temporal nature and interaction issues [70]. Numerical instabilities or gimbal lock occurs at $ {\theta _{2}}={\pm {\frac {\pi }{2}}}$ for Cardan angles and at $ {\theta _{2}}={0,\pm \pi }$ for Euler angles.

### Joint coordinate system

**Fig. 5:**
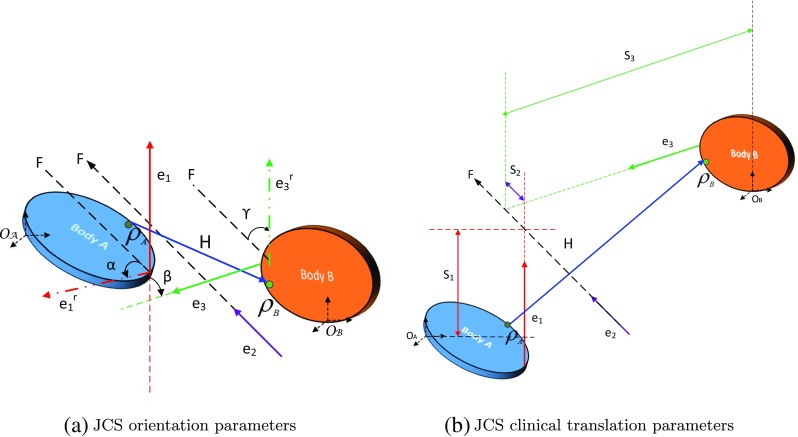
Concept of JCS and 3D motion description adapted from [71]

Inspired by the clinical movement definition, Grood and Suntay proposed the joint coordinate system in [71]. The joint coordinate system (JCS) includes six parameters, three each for rotation and translation. Importantly, the JCS description is a part of the ISB recommendation for several shoulder joints [66]. Figure 5 shows the relative kinematics problem in terms of JCS definition as given in [71].

It is known that the JCS is equivalent to the corresponding Cardan sequence [72] and can be extended to other parameterisations [71]. Similar to Euler angles, numerical singularities also occur in the JCS, at $\beta = 0$ and at $\beta = 0, S_{2}= 0$ [71]. Importantly, the JCS is sensitive to the choice of $\mathbf {e_{1}}$ and $\mathbf {e_{3}}$; an unsuitable choice can result in substantial kinematic cross-talk. The claim that JCS is “sequence-independent” in [71] is incorrect, as the specific choice of the embedded axes itself imposes a sequence effect [72].

### Denavit-Hartenberg parameters

**Fig. 6:**
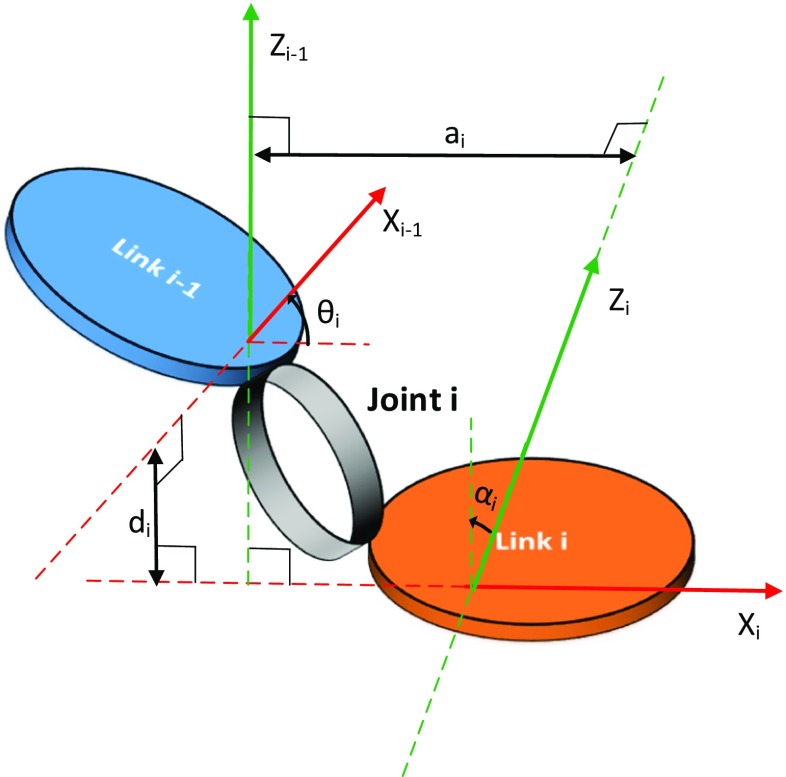
Denavit-Hartenberg parameters for joint *i* connecting link *i* and link *i* − 1

In robotics, the relative kinematics problem is often solved using the Denavit-Hartenberg (D-H) convention. In D-H parameters, the homogeneous transformation $ \mathbf {T} $ in Eq. 1 is represented by a set of four parameters. These parameters for an *i* th joint are the link length (*a*_*i*_), the link twist (*α*_*i*_), the link offset (*d*_*i*_) and the joint angle (*𝜃*_*i*_). These parameters define the geometry of link *i* with respect to link $i-1$ about a joint *i*, as shown in Fig. 6. The joints that connect these links can be of either the rotary or prismatic type. In that case, $\theta _{i}$ parameterises the rotary joint, and $d_{i}$ parameterises the prismatic joint. Because the D-H parameter definition is not unique, we follow the popular convention presented in [73]. In this case, the homogeneous transformation is given by

3 $$ {\mathbf{T}} = \!{\left[\begin{array}{cccc} \!\cos(\theta_{i})\!&\!-\sin(\theta_{i})\cos(\alpha_{i})\!&\!\sin(\theta_{i})\sin(\alpha_{i})\!&\!a_{i}\cos(\theta_{i})\!\!\\ \!\sin(\theta_{i})\!&\!\cos(\theta_{i})\cos(\alpha_{i})\!&\!-\cos(\theta_{i})\sin(\alpha_{i})\!&\!a_{i}\sin(\theta_{i})\!\!\\ \!0\!&\!\sin(\alpha_{i})\!&\!\cos(\alpha_{i})\!&\!d_{i}\\ \!0\!&\!0\!&\!0\!&\!1 \end{array}\right]} $$

In shoulder kinematics, the GH joint is often parameterised as a pure spherical joint. This effect is obtained by choosing three intersecting revolution DOF with a common origin. The D-H parameters are also equivalent to Euler angles and the JCS. Hence, numerical singularities occur. Note that the D-H parameters cannot be used in closed-loop kinematic chains as the parameter definitions become inconsistent [74].

### Other shoulder representations

Other representations are used in literature, though somewhat less prominently. The shoulder is often modelled as a combination of serial and parallel chains, which is known as a multibody or hybrid mechanism [62, 75–77]. The globe representation describes functionally important shoulder kinematics that are not restricted to the cardinal planes [78, 79]. Engin [80] used the finite helical axis (FHA) to compute the HT centrode during a humeral elevation task. Sweeping the bony links over the extreme range of motion of a joint results in an excursion cone, called a joint sinus cone [31]. An application of joint sinus cones in virtual human modelling is presented in [81].

## Review: search strategy, outline, classification and summary

We begin this section by presenting the search strategy and outline of the review, followed by the classification system used to organise the relevant literature. Subsequently, we summarise the key findings of this review.

### Search strategy

**Fig. 7:**
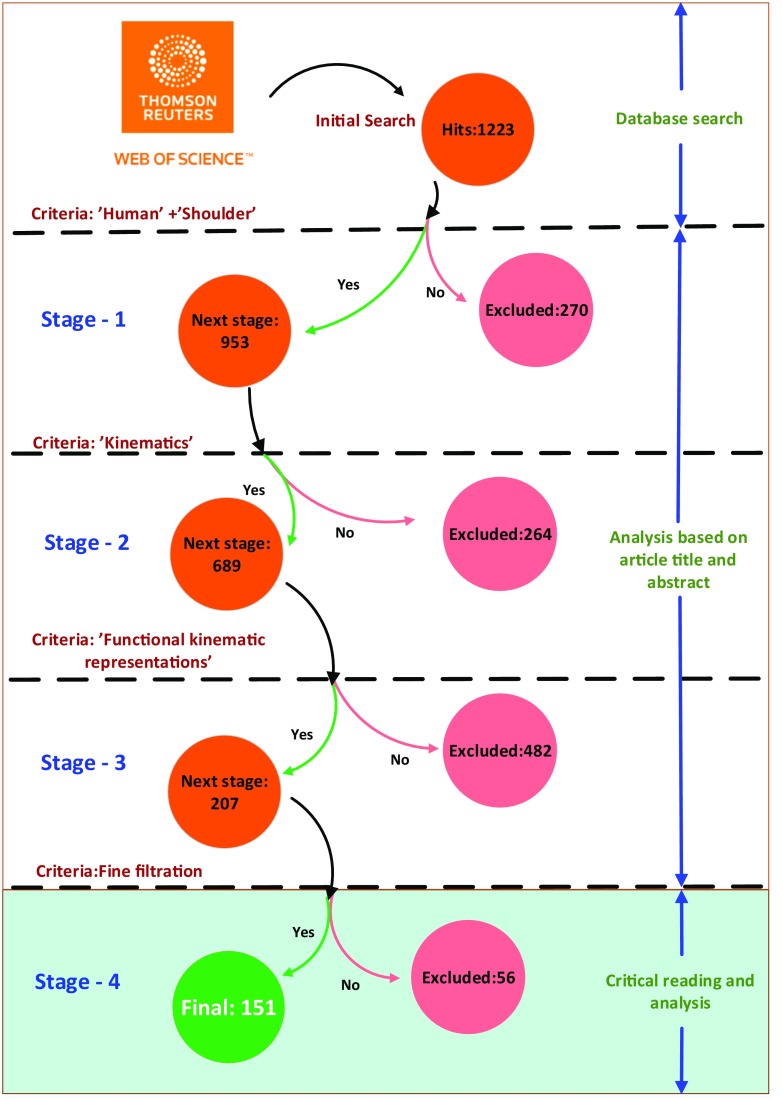
Search strategy

A systematic search based on the ISI Web of Science database was conducted on the 31 August 2017. The search keywords were “Human shoulder kinematics”, which yielded 1223 hits. Based on our review context, a four-stage detailed filtering procedure was used to narrow down the list of articles. Stages 1–3 of this filtering were based on the title and the details of the article abstract, which yielded a tentative list of 207 articles. The details of this search and inclusion strategy are presented in Fig. 7.

Recall that in the context of a functional shoulder, it is understood that clinical questions related to joint pathology, dysfunction, pain and stability are not relevant. Additionally, a few articles used healthy subjects as a control in their respective study. Using the above exclusion criteria, in Stage 4, a total of 56 articles were excluded, as they were connected to cerebral palsy (3), stroke (12), exoskeleton design (4), development disorder (6), sports (6), mechanism design (6), clinical review (1), motion classification (1), measurement (2), clinical questions (4), healthy subjects used as control (2), human-robot interaction (3), ergonomics (4) and animation (1). Additionally, one article was found to be indexed twice by the search engine and was discounted, resulting in a final list of 151 articles for review tabulation.

### Review table outline

The list of relevant papers identified in Section 5.1 is summarised in Table 1 in the Appendix. Furthermore, individual papers are arranged in rows with the columns divided into six items, namely, citation, the kinematic representation used in the study, the purpose of the study, the details of the subjects used in the study, the type of measurement instrumentation used and the activities studied.

**Table 1:** Summary of reviewed work

Literature	Kinematic representation^a^	Purpose	Subject details^b^	Measurement technique^c^	Activities^d^
Euler angles					
Robert-Lachaine [120]	SC: ZYX, AC: ZYX	3D scapulo-humeral rhythm	14 (14M $25 \pm 4$)	RFM	ABD, FLX
	GH: ZYZ, ST: ZYX				FCE, ECE
Dal Maso^∗^ [88]	GH: XZY	3D GH kinematics	4^∗∗^(4M $27-44$)	CT, RFM	ABD, FLX, AXI
Noort [99]	ST: YZX	Reliability of scapular kinematics	20 (3M, 17F: $36 \pm 11$)	IMMS	FLX, ABD
	HT: XZY/ZXY				
Seanez-Gonzalez	Euler angles	Human-machine interface	28 (12M, 16F: $24 \pm 6$)	IMMS	Cursor control
[162]					
Haering [46]	HT: ISB	DOF interaction	16 (8M, 8F: $24\pm 4$)	RFM	Series—ELE, AXI
					RAN, OVR
Massimini [85]	GH: YXZ	GH articular contact pattern	9 (4M, 5F: $26.3 \pm 2.4$)	XRF, MRI	Sc-(ELE,
					DEP, EXR)
Schwartz [128]	ST, HT: YXZ	Bilateral scapular symmetry	22 (11M: $22.4 \pm 3.6$	AMR	FLX, ABD
			11F: $22.2 \pm 1.8$,)		INR, EXR
Qin [131]	All: YXZ	Fatiguing task adaptation	20 (10F: $25.2 \pm 3.9$,	AMR	Light assembly type
			10F: $61.7 \pm 4.3$,)		task
Parel [121]	ST: YZX	Multi-centre scapulo humeral study	23 (13M, 10F, $29\pm 8 $)	RFM	FLX, EXT,
	HT: XZY, ZXY				Sc-ABD, Sc-ADD
Habechian [122]	ST: YXZ, HT: YXY,	3D scapulo-humeral kinematics	26 (M + F, $35.4\pm 11.65 $)	EMS	Static: ELE, DEP
	GH: XZY		33 (C, $9.12\pm 1.51 $)		
Worobey [100]	ST: YXZ, HT: ISB	Reliability of scapular kinematics	22 (16M, 6F:	RFM,	Static: FLX, ABD,
			50.5 ± 11.6)	Ultrasound	Sc-ABD
Lempereur [89]	GH: XZY	GH JCoR mislocation effect	11 (23.1 ± 3.36)	RFM, EOS	FLX, ABD
Zhu^∗,+^ [163]	6-DOF, Euler angles	Repeatability of shoulder kinematics	30M^⋈^, 4 (2M, 2F: 25 ± 2)	Dual XRF	ABD
Tsai [164]	YXZ	Wheelchair camber design	12 (22.3 ± 1.6)	RFM	Wheelchair
					propulsion
Shaheen [165]	GH: XZY, ST: YXZ	Scapular tracking	14M (29.4 ± 11.1)	RFM	Bilateral ABD
Phadke [90]	GH: YXY, XZY	GH rotation sequence	10 (6M, 4F: $30.3\pm 7$)	EMS	Static: Sc-ABD
Brochard [101]	ST: YXZ	3D scapular kinematics	12 (26 ± 6.18)	RFM	Static: (FLX, ABD)
Bourne [102]	HT: ISB, YZX	Scapular kinematics	8 (5M, 3F: $18-60$)	RFM	ABD, HAD, HBB,
					Reaching
Borstad [103]	ST: ZYX, HT: ZYZ	3D scapular kinematics	28 (12M, 16F:	EMS	Push-up
			25.2 ± 4.3)		
Bourne [104]	ST: YXZ, HT: ISB	Subject-specific correction factor scapular kinematics	8 (29.7 ± 4.7)	AMR	ABD, reaching, HBB, HAD
Billuart^!,∗^ [166]	XZY, 6-DOF	Role of anatomical constraints in shoulder stability	6^⋈^	XRF	ABD
Teece^+^ [167]	AC: ZYX	3D AC kinematics	8 (31-81)^⋈^	EMS	Sc-ABD
			30 (16M, 14F: $25.2\pm 3.5$)		
Sahara [168–170]	AC: XYZ, Clavicle:	3D shoulder kinematics	7M (19–30)	MRI	Static: ABD
	[171], GH: 3-DOF				
S̆enk [91]	YXY, YXZ, ZXY	Rotation sequence in GH kinematics	5 (20 − 37)	RFM	FLX, EXT, ABD, HAD, CRD^*$*^
Dayanidhi [105]	GH: XZX, ST: [172]	Scapular kinematics	15 (8M, 7F: $28.8\pm 4.3$)	EMS	Sc-ABD
			(14C: $6.7\pm 1.5$)		
Thigpen [106]	ST: YZX, HT: YXY	Repeatability of scapular	(10M: $22.9\pm 1.9$)	EMS	FLX, ABD, Sc-ABD
		kinematics	(10F: $23.7\pm 1.1$)		
Fung^!^ [107]	GH: ZYZ, ST: ZXY	Scapular and clavicular kinematics	3 (76.3 ± 6.6)^⋈^	CT, EMS	FLX, ABD, Sc-ABD
Karduna [172]	Euler angles	Effect of Euler angle sequences on ST kinematics	8 (5M, 3F: $27-37$)	EMS	Sc-ABD
Myers [108]	GH: YZX, HT: ISB,	Scapular kinematics	15 (12M, 3F: $ 29.2\pm 5.9$)	EMS	Static: ELE, DEP
	YZY				
An^!^ [92]	XZX	GH kinematics	9^⋈^	EMS	ELE
Rundquist [132]	HT: ZYZ, YXZ,	Shoulder kinematics in ADL	27 (23F, 4M: $ 22.9 \pm $	EMS	See ^◇^
	GH: YXZ, ST: ZYX		1.75)		
Zhang [173]	Euler angles	Estimation of shoulder kinematics from EMG	(6M: $ 23 \pm 1$)	RFM	Simulated drinking, FLX, EXT, ABD, ADD, hand to shoulder
Robert-Lachaine	XZY	Accuracy and repeatability of IMUs	12 (9M, 3F: $26.3\pm 4.4$)	RFM, IMMS	Material handling
[174]					
Borbély^¶^ [175]	Euler angles	Real-time inverse kinematics	OpenSim	–	Simulated
					trajectories
López-Pascual [176]	YXY, XZY	Reliability of HT angles	27 (14M, 13F: 38.2 mean)	RFM	Arm lifting
Denavit-Hartenberg parameters					
Cortés [14]	D-H (seq: [177])	Kinematic estimation for exoskeleton	4 (4M:34 (*mean*))	RFM	–
Rosado^*%*^ [178]	3-DOF, 5-DOF	Reproduction of human-like movements	–	Kinect	Circular rhythmic motion of hand
El-Gohary [179]	D-H parameters	Tracking shoulder angle using IMMS	8 (2 groups)	RFM, IMMS	ABD, ADD, FLX, EXT, Reaching doorknob, touching nose
Zhang [180]	3-DOF, D-H parameters	Measurement of limb kinematics using IMMS	4 (nil)	RFM, IMMS	Arbitrary movement
Lv^¶,‡^ [181]	5-DOF, D-H parameters	Biomechanics based life like reaching controller	–	–	Reaching movement
Jarrasse [13]	3-DOF, D-H parameters	Avoid hyperstaticity when in human-exoskeleton interaction	Nil	Optical encoder	Trace a metallic wire
Kundu [182]	3-DOF, D-H parameter	3D analysis in ergonomics	5M (23.8 ± 1.79)	RFM	Lever manipulation
Klopc̆ar and Lenarc̆ic̆^*%*,‡^	5-DOF	Arm reachable workspace	1F (25)	–	Random
[183–185]					
Schiele [144]	5-DOF, D-H parameters	Ergonomic exoskeleton design	4M (nil)	AMS	ABD, FLX, EXT, DRI, HAC, BAW
Klopc̆ar [186]	4-DOF	Bilateral and unilateral shoulder girdle kinematics	10 (5M, 5F: $24.8\pm 1.4$)	AMS	ELE^∙^
Lenarc̆ic̆^¶^ [77]	D-H	Humanoid shoulder models	–	–	Humeral pointing
Liu [187]	D-H	Anthropomorphic motion generation	–	Kinect	Random movements
Kashima^*%*^ [188]	D-H	Biomimetic control of robot	1	RFM	Straight and curved hand trajectories
Joint coordinate system/ISB					
Laitenberger [189]	SC, AC: ISB	Multibody analysis	15 (5F: $24\pm 2$	RFM	FLX, EXT, ABD
	GH: ZYZ		10M: $27\pm 6$)		ADD, CRD
El-Habachi^∗^ [83]	ST: ISB	Multibody analysis	6 (6M: $22.67\pm 1.97$)	EMS	Static: ABD
	GH: Euler (XZY)				
Srinivasan [190]	ISB	Quantify motor variability	14 (14F: 20-45)	EMS	Pipetting
Charbonnier^∗^ [52]	GH: JCS (XZY)	3D GH kinematics	6 (6M :39.6 ± 7)	MRI, RFM,	FLX, ECE
				and XRF	
Xu [65]	ISB	Regression-based 3D shoulder	38 (19M, 19F	AMR	118 static postures
		rhythm	32.3 ± 10.8)		
Bolsterlee^*%*^ [191]	ISB	Simulation of scapula and clavicle	5 (3M, 2F, $29.2\pm 2.3 $)	AMR	FLX, ABD
Matsuki [41]	ISB	Comparison of bilateral clavicular	12M (20 − 36)	XRF, CT	Sc-ABD
		kinematics			
Xu [142]	ISB	Effect of external frame devices in	6 2M, 4F (33.7 ± 11.3)	AMS	118 static postures
		shoulder kinematics			
Roren [109]	ISB	Reliability of 3D scapular	13 (7M, 8F $30.2\pm 9.4$)	EMS	FLX, ABD, HAC,
		kinematics			BAW
Prinold [110]	GH: ISB, ST: YXZ	Effect of speed on scapular	16 (M, $25\pm 2$)	RFS	FLX^*ø*^, Sc-ABD^*ø*^
		kinematics			
Newkirk [143]	ISB	Quantifying gross shoulder motion	20 (10M, 10F, $25.3\pm 1.4$)	EMS, AMR	Free ROM task
			17 (11M, 6F, $27.6\pm 3.2$)		
Pereira [133]	JCS	Compensated HT kinematics	6 (3M, 3F: $23.8\pm 0.98$)	RFM	Turning doorknob,
					using Screwdriver,
					answering phone,
					feeding, take and
					insert card
Hagemeister [192]	JCS	Axis alignment in shoulder	5 (20 − 37)	RFM	Sc-ABD$^{{\blacktriangle \blacktriangle }}$
		kinematics			
Vandenberghe [134]	ISB	Factors affecting 3D reaching	10 (6M, 4F: nil)	AMR	Reaching^∇∇^
Kedgley^!^ [111]	GH: ISB	Reliability of scapular coordinate	11^⋈^	CT, XRF	15 postures
		system definition			
Crosbie [112]	ISB	Scapular kinematics in a lifting task	45F (20 − 80)	EMS	FLX, bimanual
					lifting^♣♣^
Oyama [113]	ISB	Scapular and clavicular kinematics	25 (14M, 11F $23.2\pm 2.4 $)	EMS	Retraction exercise
Rezzoug [193]	3-DOF, ISB	Estimation of 3D arm motion	10M(26 ± 5)	EMS	Calibration gestures
Lovern [57]	ISB	GH kinematics in ADL	5 (2M, 3F $23\pm 1$)	RFM	ABD, Sc-ABD, FLX,
					10 ADL^§^
Braman [93]	ISB, GH: XZY	GH and ST kinematics	12 (7M, 5F: $29.3\pm 6.8$)	XRF, EMS	Reaching
Amadi^∗,¶^ [94]	JCS	GH physiological kinematics	F	VHP	Static: FLX, ABD
Forte [58]	ISB	3D scapular kinematics and	11 (26.7 ± 5.2)	RFM	Quasi-static: ABD^♣♣^
		scapulo-humeral rhythm			
Chapman [194]	ISB	Unconstrained joint position	23 (13M, 10F: $21.7\pm 4.8$)	EMS	ELE$^{{\bigstar }}$
		sense task			
Jacquier-Bret [135]	ISB	Reach-grasp adaptation	29M(26.2 ± 5)	RFM	Reaching$^{{\bigstar \bigstar }}$
Langenderfer [195]	ISB	Effect on landmark location in	11 (6M, 5F: $24.6\pm 6.1$)	EMS	Sc: ABD (30^*o*^ − 90^*o*^)
		shoulder kinematics			
Fayad [114]	ISB	3D scapular kinematics	30 (14M, 16F: $24.7\pm 4.7$)	EMS	FLX^*øø*^, ABD^*øø*^
Levasseur $^{{!}}$ [51]	ISB	Effect of axis alignment on	8 (59 − 87)	EMS	Sc-ABD
		kinematics			
Lin [196]	ISB	Humeral kinematic measurements	14 (7M, 7F: $22.6\pm 4.8$)	EMS, IMMS	ELE, INR
Scibek [197]	ISB	Repeatability of shoulder	11 (5M, 6F: $21.44\pm 1.42$)	EMS	FLX, ABD, Sc-ABD
		kinematics			
Robert-Lachaine	ISB, MVN	Validation of IMU	12 (9M, 3F: $26.3\pm 4.4$)	RFM, IMMS	Material handling
[198]					
Nicholson^!^ [119]	ISG [199]	3D scapular orientation	12 skeletons	RFM, RSA	Various scapular orientations
Tse [200], McDonald	ISB	Shoulder fatigue during repetitive	12 (20–24)	RFM	Fatiguing protocol
[201]	work				
Hernandez [202]	ISB	Evaluating upper limb force	10 (28.5 ± 3.9)	RFM	Elbow FLX-EXT
		capacities			
Pirondini [203]	ISB	Effect of exoskeleton on movement	6 (5M, 1F: $26.5\pm 3.4$)	RFM, ALEx	Reaching with and
		execution		exo	without exo
Miscellaneous					
Vanezis [204]	Jaspers’ [205]	Inter-session reliability	10 (4F, 6M: $13.6 \pm 4.3$)	RFM	4 RGT, HCS, HBP
					DRI, THR
Dounskaia [206]	3-DOF	Interpreting joint control pattern	11 (7M, 4F: $24 \pm 4$)	EMS	Free stroke drawing
					task
Lempereur^*#*^ [115]	–	Scapular motion analysis review	–	–	–
Yan [207]	[208]	Shoulder compatible exoskeleton	6 (25.17 ± 3.6)	RFM	FLX, ABD
Cutti [209]	ISEO	PBIs of normal scapular kinematics	111 (38 ± 14)	IMMS	FLX, EXT, ABD
					ADD, PRO, RET
					MER, LAR, ANT, POT
Ricci [210]	–	Protocol for typically developing	40C (6.9 ± 0.65)	IMMS	ABD, ADD
		children			FLX^†^, EXT^†^
Pierrart [211]	–	Dynamic-MRI for shoulder	4 (1M, 3F: 30-45)	MRI	ABD
		kinematics			
Lenarc̆ic̆^*#*^ [84]	–	Computational kinematics	—	–	Shoulder example
Gaveau [130]	Planar	Gravity vector in movement	10M (23.8 ± 1.8)	RFM	FLX, EXT
		planning			
Xu [127]	Ball and socket	Effect of age on inter-joint synergies	18 (9F, $25.6\pm 3.9$)	AMR	Light assembly task
			(9F, $61.8\pm 4.5$)		
El-Habachi^¶^ [212]	Parallel mechanism	Sensitivity of multibody shoulder	Visual human	–	Free ROM task
		parallel mechanism	project (VHP)		
Simoneau^¶^ [126]	Planar angle	Role of trunk rotation in reaching	–	–	Reaching
Pontin [213]	Planar angle and	Scapular positioning	30 (13M, 17F: $24.5\pm 7.1$)	RAD	Static examination
	distance				
Mallon^*#*^ [50]	Group model	GH motion and Codman’s paradox	–	–	24 static positions
Xu$^{{\blacktriangle }}$ [214]	Rotation matrix and	Mapping between various scapular	13 (9M, 4F, $41\pm 14$)	CT	
	translation vector	coordinate systems			
Xu$^{{\blacktriangle }}$ [215]	Matrix transformation	Mapping between Holzubar	–	–	–
		[87] and ISB [66]			
Jackson [53]	15-DOF	Introduction of reference position in	15M (25 ± 4)	RMS	FC-FLX, FC-ABD,
		ISB [66]			EC-FLX, EC-ABD
Kim [136–138]	3-DOF, exponential	Redundancy resolution in	10 (8M, 2F, 32 avg)	AMR	Reaching^♣^, grasping^♣^,
	map	upperlimb exoskeleton			peg-in-hole^∇^
Massimini [54]	Translation	Quantify GH joint kinematics	5M (26 ± 4)	Dual XRF,	Static: ABD
				MRI	
Izadpanah [216]	Length	GH ligament kinematics	13 (6M, 7F: $25\pm 2$)	MRI	Static: ABD
Massimini^∗,!^ [55]	6-DOF	Scapula and humerus coordination	30M^⋈^	Dual XRF,	ABD, ADD, INR,
				CT	EXR
Amadi^*#*,¶^ [56]	Mobile square	GH kinematics	VHP	–	FLX, ABD, ADD
	window				
Lee^!,∗^ [95]	Translations,	3D GH contact kinematics	6 (1M, 5F: $49-97$)^⋈^	Microscribe	NR, EXR
	asymmetric features				
Yano [116]	Planar angles	3D scapular kinematics and	21 (17M, 4F: $18-27 $)	AMR	Sc-ABD
		shoulder rhythm			
Yang^+^ [96]	Length	Role of GH ligaments	5 (2M, 3F: 60-96)^⋈^	CT, MRI	Static: ABD
			7M (19-30)		
Lovern [117]	–	Scapular tracking	10 (6M, 4F: $27.5\pm 5.1$)	RFM	Static: FLX, ABD
Yang^¶^ [217, 218]	Euler angles, D-H	Analytical mapping between Euler	–	–	–
		angles and D-H parameters			
Folgheraiter [219]	Parallel mechanism	Wearable exoskeleton	1M	–	EXT
Kon^∗^ [123]	6-DOF	Effect of load on scapulo-humeral	10 (8M, 2F: $27-38$)	XRF, CT	ABD^♣♣^
		rhythm			
Amadi^*#*^ [118]	–	Definition of scapular coordinate	16 (57 − 79)	CT	–
			system		
Boyer [59]	6-DOF	GH contact kinematics	5M (26 ± 4)	Dual XRF,	ABD^*$xx*−*xx*^
				MRI	
Berman [33]	Motors/screw axis	3D movement planning	4M (18-32)	AMR	Reaching^*$$xx*−*xx*^
Hill^*#*^ [34]	–	GH clinical kinematic model review	–	–	–
Cutti [220]	D-H,	Shoulder kinematics using IMMS	1M (23)	IMMS, RFM	See^§§^
	ST: YZX, HT: XZY				
VanAndel [4]	ISB, HT: globe	3D kinematics in functional task	10 (6M, 4F: $28.5\pm 5.7$)	AMS	See^††^
Illyás [221]	See: [222]	Shoulder kinematics using	50 (32M: $28.1\pm 5.1$)	Ultrasound	
		ultrasound	(18F: $24.6\pm 6.12$))		
Bobrowitsch^¶^ [223]	Shape analysis, ISB	Humeral kinematics	Volunteer	MRI	–
Dennerlein [224]	See [225]	Contribution of shoulder in typing	6 (4M, 2F: 30-41)	AMS	Shoulder only typing
Bey $^{{!}}$ [97]	Model-based tracking	GH kinematics	3 (89 ± 6.2)^⋈^	RSA, CT	ABD, FLX, EXR
Sapio^¶,∗^ [226]	Holzbaur [87]	Control of a humanoid and realistic	–	–	Humerus pointing
		shoulder model			
Klein-Breteler^*%*^ [38]	Quaternion	3D object manipulation	15 (5M, 10F: $24.7\pm 3.6$)	RFM	Center-out-task,
					cylinder rotation
Kang [227, 228]	3-DOF	Kinematic redundancy	4	AMS	Reaching movement
Magermans [139]	ISB, globe	3D activities of daily living	(24F: $ 36.8\pm 11.8$)	EMS	See^∙∙^
Holzbaur^¶^ [87]	GH: 3-DOF, [229]	Musculoskeletal model for surgery	50th percentile male	–	FLX, EXT,
					ABD, ADD, INR, EXR
Rosen [140]	3-DOF	ADL analysis for 7-DOF exoskeleton	1	RFM	See^◇^
Endo [230]	Planar	Effect of age on ST kinematics	12	RAD	Cylindrical handle,
					load lifting
Novotny^*#*^ [231]	Rate Euler	Measuring axial rotation	Gimbal mechanism	EMS	INR, EXR
Prokopenko [141]	6-DOF	Accuracy of arm model	6 (4M, 2F: $26-52$)	EMS	ABD, ADD,
					FLX, EXT,
					INR, EXR^◇◇^,
					reaching
Baerlocher^*#*^ [232]	Angle axis	ROM and limits in a ball and socket	–	–	–
		representation			
Cheng^*#*^ [148]	3-DOF	Spherical rotation coordinate	–	–	–
		systems			
Maurel^¶^ [81]	Joint sinus cones	Realistic shoulder animation	–		–
Novotny^¶,!^ [98]	6-DOF	GH ligament kinematics	1^⋈^	–	ABD, EXR
Pascoal [124]	ST: YZX, humeral	Effect of load on SHR	(30M: $ 23.8\pm 2.8$)	EMS	FLX, ABD, Sc-ABD
	angle				
Kamper^¶^ [233]	Loci	Neural kinematic strategies	–	–	Reaching
Romkes [234]	Gutierrez [235]	Effect of gait on upper body	20 (10M, 10F: $24.9\pm 2$)	RFM	Arm swing at
		kinematics			different gait speeds
Salmod [236]	Planar angle	Movement smoothness	10 (5M, 5F: $23\pm 3$)	EMS	Horizontal reaching
					at different speeds
Florian [237]	Planar angle	Fatiguing task	17 (25.1 ± 0.5)	IMMS	Ballistic reaching
Togo $^{{\%}}$ [238]	Planar angle	Human-like joint coordination	8M	RFM	Tracking task
Lorussi [125]	Bi-articular	Shoulder rhythm	5	IMMS, RFM	FLX, ABD
Krishnan [157]	Hybrid twists	Singularity-free functional HT	(4M: $24\pm 3.36$)	RFM	ABD, ADD, FLX,
		kinematics			EXT, ELE, DEP

^a^ ISB refer to [66]; JCS: joint coordinate system; ISEO refer to [220]; D-H: Denavit-Hartenberg parameters

^b^ F female; M male; C children

^c^ RFM: retro-reflective markers; EMS: electromagnetic; CT: computed tomography; MRI: magnetic resonance imaging; XRF: X-ray fluroscopy; IMMS: inertial and magnetic measurement system; AMR: active marker; EOS: low dose stereo radiographic imaging; RAD: radiography; RSA: radiostereometric analysis

^d^ FLX: flexion/anteflexion; EXT: extension; ABD: abduction; ADD: adduction; CRD: circumduction; RGT: reach to grasp task; HCS: hand to contralateral shoulder; HBP: hand to back pocket; DRI: drinking; THR: throwing; FCE: full can exercise, ECE: empty can exercise; AXI: axial rotation with zero elevation; RAN: random; OVR: overall; Sc: scapular plane; ELE: elevation; DEP: depression; EXR: external rotation; INR: internal rotation; PRO: scapular protraction; RET: scapular retraction; MER: medial rotation; LAR: lateral rotation; ANT: anterior tilt; POT: posterior tilt; Sc: scapular plane; HAC: hair combing; BAW: back washing; FC: full can; EC: empty can; HAD: horizontal adduction; HBB: hand behind back; HAD: horizontal abduction

*Classification indices*

^*^ Realistic study; $^{!}$In vitro study; $^{+}$In vitro + in vivo study; $^{\P }$In silico study; $^{\#}$Not an in vivo study; $^{\%}$In vivo + in silico study; $^{\dagger }$Forward approach; *Special notes*; $^{\ddagger }$With and without holding a bar; $^{\o }$At varying speeds slow, normal and fast; $^{**}$One data excluded; $^{\bowtie }$Details of cadaver; $^{\blacktriangle }$Cannot be classified as a humanoid or realistic study; $^{\clubsuit }$Both constrained and unconstrained; $^{\nabla }$Only one subject; $^{\blacktriangle \blacktriangle }$Three different palm orientations neutral, internally, externally rotated; $^{\nabla \nabla }$Different reaching heights and widths; $^{\clubsuit \clubsuit }$Both with and without load; $^{\S }$Reach to opposite axilla, reach to opposite side of neck, reach to side and back of head, eat with hand to mouth, eat with spoon, drink from mug, answer telephone, brush opposite side of head, lift block from shoulder level and overhead refer [57]; $^{\bigstar }$Activity performed with visual cues and trunk upright and tilted 45^∘^; $^{\bigstar \bigstar }$Reaching without, with a medium and large obstacles; $^{\$}$With axial rotations: neutral, maximal internal rotation, maximal external rotation; $^{\$\$}$Radial and frontal plane reaching; $^{\S \S }$FLX, EXT, ABD, ADD, INR (neutral and 90^∘^ ABD humerus), EXR (neutral and 90^∘^ ABD humerus), hand to nape, hand to top of head; $^{\ddagger \ddagger }$FLX, EXT, ABD, ADD, INR (90^∘^ ABD humerus), EXR (90^∘^ ABD humerus), hand to contralateral shoulder, drinking, combing hair, hand to back pocket; $^{\o \o }$Self-selected slow, fast pace, three static positions; $^{\bullet }$Four different anatomic planes during unilateral and bilateral shoulder motion; $^{\bullet \bullet }$FLX, EXT, ABD, ADD, INR (90^∘^ scapular abduction), HBB, eating with spoon, combing hair, lifting task, wash axilla, overhead reaching; $^{\diamond }$FLX, EXT, ABD, ADD, INR, EXR, 24 ADLs, see [140]; $^{\diamond \diamond }$All movements performed passively; ^◇^Washing back, feeding, combing, reaching overhead, washing contralateral axilla

Because the majority of studies use Euler angles, they have been indicated by the relevant sequence only. The joints of interest in the respective studies have been indicated by appropriate abbreviations presented in Section 2.1.

Because the statistical validity of any study depends on the number of subjects involved, we decided to highlight the subjects used in the reviewed articles by indicating the total number of subjects in the study, followed by their details: male (M), female (F), child (C) and their respective age distributions.

The method of human motion tracking used is crucial. Therefore, we have also tabulated the variety of measurement techniques used in the reviewed articles. Additionally, the different movements in the study have been summarised. Let us proceed to examine the classification system used to organise the literature.

### Classification scheme for reviewed papers

From Section 4, it is clear that there is a large diversity among the kinematic representations used in the shoulder kinematics literature. Although it is challenging to classify the available literature, we have proposed a three-point classification strategy, which is discussed below.

#### Realistic or humanoid representation

What is the real nature of shoulder motion? The answer to this simple question is not straightforward, because the definition of reality is both context- and purpose-specific in nature. A recent survey and experimental study provides a detailed summary on the use of multibody methods in upper limb kinematics [62, 82]. As discussed in Section 2, the functional shoulder motion consists of simultaneous rotations and translations. Because HRI is situated in real world, it is important that the models used in cHRI are realistic [22]. Therefore, in the context of high-reliability HRI, we classify the studies that represent the shoulder joint as a ball-and-socket joint as a *humanoid*. In contrast, the studies that treat the shoulder otherwise are classified as *realistic*. Additionally, following the recommendation by El-Habachi et al. [83], the studies that treat the shoulder as a closed-loop kinematic chain are considered *realistic*. Because the majority of the reviewed papers use a humanoid approach in parameterising human shoulder kinematics, we indicate realistic studies by the footnote marker (*).

#### Forward or inverse kinematics

In shoulder kinematics, finding the humeral position given the individual joint configurations poses the *forward* problem. Note that the forward problem has guaranteed uniqueness [8, 84]. Forward studies commonly extend our understanding of individual joint contributions and our knowledge of the human arm-reachable workspace. In contrast, finding the joint variables from the kinematic measurements poses the *inverse* problem. Note that this challenging problem has no unique solution [8]. In both cases, the kinematic inference is based on the representation of choice. Note that because there are only a handful of forward studies in shoulder kinematics, we denote them using the footnote label (‡).

#### Biological context

Traditionally, the anatomical understanding has emerged from studies based on human cadavers, which are known as in vitro studies. However, it is well known that in vitro studies do not replicate the properties of any living shoulder [41, 59, 85, 86]. Studies based on living humans are called in vivo research [86]. Increased computational power has enabled numerical and simulation studies of the musculoskeletal system, which are known as in silico studies [86]. They play an important role in investigations that would be otherwise impossible to measure or quantify or would require an invasive approach [49]. An example of an in silico study in the context of musculoskeletal surgery is given in [87]. In silico models will play a significant role in future research because cadaveric studies are expensive and pose ethical challenges [50].

Although the classification system is quite straightforward, in reality, different studies have used all the above three combinations to varying degrees. The majority of the reviewed papers fall under the purely in vivo category. Therefore, we denote the in vitro studies by (!), the in silico studies by (¶), the combination of in vivo and in vitro studies by (+ ), the combination of in vivo and in silico studies by (%) and not an in vivo study by (#).

### Review summary

**Fig. 8:**
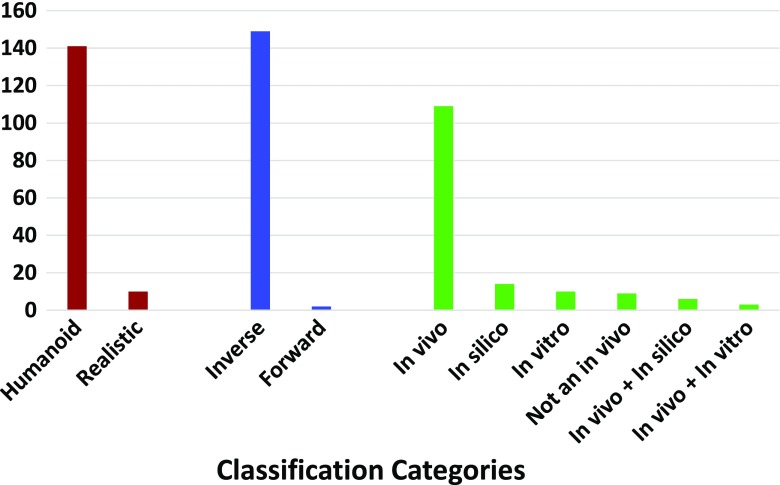
The histogram shows the number of reviewed articles classified according to the categories presented in Section 5.3. The three different colours respectively represent the three literature classification categories

In Table 1 in the Appendix, the entries have been grossly grouped according to the kinematic representations used: Euler angles, D-H parameters, joint coordinate system and other. Out of the 151 reviewed studies, Euler angles were used by 37 studies, whereas JCS was used by 35 studies. The popularity of these representations might be due to the intuitive nature of both of these representations and their closeness to the clinical definition. Figure 8 presents the results of the literature classification of our survey. Note that the majority of the reviewed papers are in the humanoid, inverse kinematics and in vivo categories.

We could also see that the purpose of the various studies is diverse. The most frequent ones are GH kinematics [34, 50, 52, 55–57, 59, 85, 88–98], scapular kinematics [55, 99–119] and shoulder rhythm [58, 65, 116, 120–125]. Several studies in shoulder kinematics have been interested in analysing the effects of various factors on kinematics, including age [112, 122, 126, 127], load [58], dominance [57, 128, 129] and gravity [130].

**Fig. 9:**
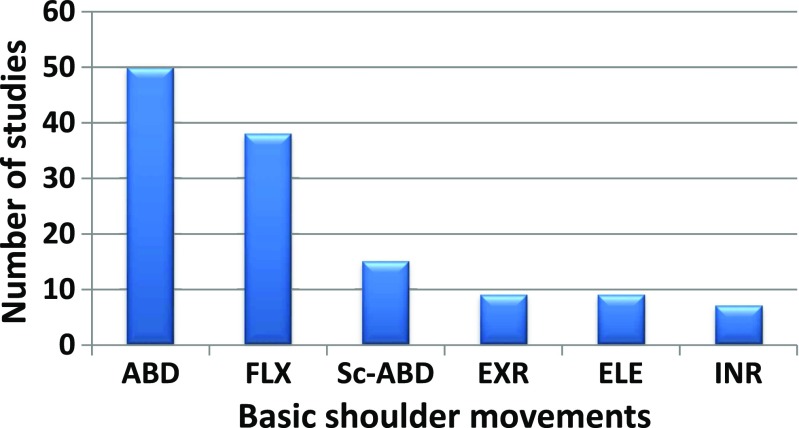
A summary of major shoulder movements in the literature. Note that only the movements that occur with a frequency greater than five are considered here. The notations are as follows: ABD—abduction, FLX—flexion, Sc-ABD—adduction in the scapular plane, EXR—external rotation, ELE—elevation and INR—internal rotation

The frequency of the basic shoulder movements in the reviewed literature is presented in Fig. 9. This histogram shows that shoulder abduction and flexion are frequently evaluated in kinematic analysis. They are followed by abduction in the scapular plane, which is seldom used in daily life. The preference for the abduction movement might be due to the ease of measurement and the almost ball and socket behaviour of the GH joint during the initial phases of abduction. However, internal/external rotation and elevation were used less frequently. The reason might be connected to the presence of STA, which might pose initial measurement challenges. In contrast, the abduction movement generates the least STA. Additionally, several studies [4, 33, 38, 126, 131–141] took an interest in analysing ADL.

## Discussion

Although the ISB recommends the Euler YXY sequence for reporting HT kinematics, there is a lack of consensus on the best rotation sequence [142]. In 3D-ROM analysis, it is a common practice to extrapolate the planar ROM, but it is now known that such analysis leads to 60% non-physiological poses [46].

Because the GH joint has the largest ROM in the shoulder, it is a common practice to approximate the shoulder kinematics to that of the GH joint. Therefore, a common assumption prevails that the GH joint is equivalent to a ball and socket joint, which we will challenge below.

### Ball and socket assumption

Fundamentally, the ball and socket assumption neglects the role of joint structures such as ligaments [34, 94], translations [54], joint asymmetries [95] and the role of the girdle [14, 50, 143]. This assumption only holds for a small ROM and deviates significantly during a large ROM [144]. Therefore, it can be argued that this approach is an inappropriate use of reductionism. Hence, the validity of this assumption in high-reliability applications must be reconsidered.

Thus, it can be argued that the GH joint alone cannot completely capture the function of the shoulder articulation. Moreover, mathematical simulations aimed at comparing the pure GH and the whole girdle workspace have shown significant kinematic differences [77]. Importantly, as we have emphasised before, even small ROM contributions from joints other than the GH are important and significantly affect the end goal of an activity [139]. However, this simplification remains popular due to the ease of clinical interpretation [50].

### Kaltenborn’s convex-concave rule

Approximating the shoulder articulation by lower kinematic pairs (see Section 4) is based on the assumption that the articulation follows the convex-concave principle [2]. This principle describes the relation between a joint’s congruency and its kinematics [47]. The principle is stated as: “A concave joint surface will move on a fixed convex surface in the same direction the body segment is moving. On the other hand, a convex joint surface will move on a fixed concave surface in the opposite direction as the moving body segment [47].” Importantly, several experimental studies have shown that the convex-concave rule is violated by the shoulder even for simple movements [145, 146]. Moreover, the validity of this reductionism in turn depends on the joint curvature [147]. If the shoulder articulation does not follow this rule, the error we commit in assuming a lower kinematic pair is significant. Therefore, it is important to reconsider this incorrect usage of reductionism in the context of high-reliability applications.

### A note on common kinematic errors

1. The spherical coordinate system presented in [148] uses a combination of rotations about the local and global axes that is not recommended [149]. Although the representation can be physically intuitive, note that spatial rotations are path-dependent even if their initial and final positions are the same [38]. Therefore, it is mathematically incorrect to claim “sequence independence”. Such a situation can be avoided by precisely and explicitly describing the steps, rotation vectors, axis orientations, reference frames and order of rotation [149].
2. Another common erroneous usage of rotation angles is in the computation of ROM, when researchers treat them as vectors. Importantly, this approach can result in the misinterpretation of phenomenon [150]. Instead, it is recommended to use the difference of rotation matrices to extract the ROM [150].

## Moving towards high-reliability human-centric kinematic models

Now, we ask whether the existing shoulder kinematic representations are suitable for high-reliability HRI. Based on our review, it is clear that humanoid representations (see Section 5.3.1) are the most commonly preferred ones in shoulder kinematics. Undoubtedly, this approach represents a highly simplified situation. Such simplifications make error due to representational mismatch unavoidable. Moreover, the non-linear and time-varying nature of kinematics exacerbates this situation, thereby undermining the very purpose of these representations. This computational challenge is even more daunting in the case of the human-centric models that form the basis of HRI [12, 48]. For successful robot-assisted rehabilitation, the robot needs to somehow incorporate the knowledge of the patient’s health that emerges from functional understanding.

Importantly, existing clinical scales in rehabilitation have been criticized to be low in validity, reliability and sensitivity [28]. Moreover, for such an analysis, it is time consuming and expensive to collect data. Alternatively, a robot-based or sensor-based solution can provide high-quality data; thereby, many of the above limitations can be overcome [28]. If properly designed, robot-based rehabilitative solutions can simplify the patient’s assessment [28]. With highly reliable rehabilitation technology, even the group size for the randomised control trials (RCTs) can be reduced [28, 151]. Eventually, we will be able to minimise the high costs involved in running RCTs [152]. Moreover, highly reliable measurements will enhance the confidence in the interpretation of clinically relevant treatment effects [153]. Therefore, improving the measurement reliability will have a significant impact on the future of both rehabilitation research and practice [151, 152].

### Meeting the high-reliability computational challenge

As we have mentioned before, meeting this challenge remains an open research question. Therefore, for possible answers, we might have to look beyond current approaches in biomechanics, robotics and human motor control [48]. Therefore, we suggest possible ways to meet this computational challenge.

#### Embracing redundancy

Biologically, redundancy is advantageous and highly desirable [135]. However, minimalist parameterisations such as the Euler angles are widely preferred, as is evident from our review (see Table 1 in the Appendix). Mainly, these representations cannot effectively capture this inherent redundancy in upper limb kinematics [34, 135]. Mathematically, minimal representations using three parameters are prone to numerical singularities [149], which are undesirable in high-reliability applications.

One of the strongest criticisms against minimalism is that the computational power of the human brain is immense. Therefore, controlling multiple DOF should not pose any problem to the human brain [154]. Although simplicity and lower levels of abstraction are highly desirable traits in a model, it can be argued that such an approach provides only limited understanding in applications such as robot-assisted rehabilitation [155]. Non-minimal representations, however, need to be backed by highly reliable measurements [34]. Moreover, complexity in mathematical representation leads to an increased level of abstraction, resulting in interpretation difficulties [34]. These points are important limitations of redundant approaches. However, the issue of redundancy holds the key to the high-reliability computational challenge. Therefore, we believe that new kinematic representations might present a possible answer to this challenge.

#### Incorporating the translations well

As can be seen in Section 6.1, the shoulder function is mathematically approximated by a ball and socket joint. In fact, it is a challenge to encode the translations using the clinical movement definition [34, 50], which motivates the widespread use of this approximation. Through a slight change in the mathematical perspective, however, it is possible to handle the simultaneous rotation and translation with ease.

Mathematically, the order in which the homogeneous transformation matrix is decomposed into rotation and translation has important implications, as this decomposition is not commutative (see Eq. 1). Generally, the homogeneous transformation is decomposed following the displacement first and rotation second rule. This rule results in the *passive* kinematic interpretation of the movement [156]. In contrast, reversing this order of interpretation results in an active interpretation [156]. Importantly, active interpretations embed translations effortlessly without the need of any explicit body-fixed frame. Although active representations are simpler, their clinical interpretation is still difficult. Because existing clinical interpretation is inherently passive. Currently, it is challenging to switch between active and passive kinematic representations [157].

#### Emphasis on functional understanding

Thus far, current approaches in shoulder kinematics fall under the umbrella of deterministic models, especially if they are hierarchical in nature. In hierarchical models, the mechanical quantities involved in the first level must completely determine the factors included in the next higher level [158]. Conversely, the performance of these models worsens in the presence of joint translations and irresolvable information on axial rotations [159, 160]. On a similar note, a common criticism exists that the hierarchical approach does not contribute to functional understanding [161].

An alternative to this existing approach is the 6-DOF approach, which can potentially address many of the abovementioned shortcomings of the hierarchical models. The 6-DOF models can ensure kinematic decoupling, lower error propagation and better tracking of non-sagittal joint rotations [159]. However, the 6-DOF marker set is sensitive to noise [159]. Despite this shortcoming, the 6-DOF models have the potential to be used in high-reliability HRI because such an approach would enhance functiona l understanding.

Movement kinematics forms the cornerstone of today’s neuromuscular modelling. Therefore, kinematics will be crucial in addressing many open problems in neuromuscular modelling: development of universal biological joint, rigorous validation of developed models, and not limited to automating movement analysis [86]. From the perspective of robot-assisted rehabilitation, future cognitive models must be able to answer the “When to assist and what to assist?” question [21].

## Conclusions

In conclusion, we have highlighted the importance of shoulder articulation in daily life, and we have systematically searched and compiled the existing literature on human shoulder functional kinematics. We have thereby successfully highlighted important gaps in our current knowledge with respect to the high-reliability computational requirement, in applications such as robot-assisted rehabilitation. The findings of our review were reframed in the light of this high-reliability computational challenge. It was found that current approaches in different disciplines cannot meet this challenge. Possibly, this challenge could be met by new kinematic representations that are redundant, active and that emphasise on functional understanding. Therefore, more efforts are needed in this direction. Only then can robot-assisted rehabilitation reach its full potential.
